# Oxidative Stress Associated with Chilling Injury in Immature Fruit: Postharvest Technological and Biotechnological Solutions

**DOI:** 10.3390/ijms18071467

**Published:** 2017-07-08

**Authors:** Juan Luis Valenzuela, Susana Manzano, Francisco Palma, Fátima Carvajal, Dolores Garrido, Manuel Jamilena

**Affiliations:** 1 Departamento de Biología y Geología, Campus of International Excellence (ceiA3), CIAIMBITAL, Universidad de Almería, 04120 Almería, Spain; jvalenzu@ual.es (J.L.V.); manzano@ual.es (S.M.); 2 Departamento de Fisiología Vegetal, Facultad de Ciencias, Universidad de Granada, Fuente Nueva s/n, 18071 Granada, Spain; fpalma@ugr.es (F.P.); fclintu@correo.ugr.es (F.C.); dgarrido@ugr.es (D.G.)

**Keywords:** chilling injury, immature fruit, oxidative stress

## Abstract

Immature, vegetable-like fruits are produced by crops of great economic importance, including cucumbers, zucchini, eggplants and bell peppers, among others. Because of their high respiration rates, associated with high rates of dehydration and metabolism, and their susceptibility to chilling injury (CI), vegetable fruits are highly perishable commodities, requiring particular storage conditions to avoid postharvest losses. This review focuses on the oxidative stress that affects the postharvest quality of vegetable fruits under chilling storage. We define the physiological and biochemical factors that are associated with the oxidative stress and the development of CI symptoms in these commodities, and discuss the different physical, chemical and biotechnological approaches that have been proposed to reduce oxidative stress while enhancing the chilling tolerance of vegetable fruits.

## 1. Postharvest Technology and Physiology of Immature Fruits

### 1.1. Immature Fruits

A number of immature vegetable-like fruits are produced by dicotyledonous species of great economic importance, within the families *Cucurbitaceae* (pumpkins, cucumbers, zucchini, bitter gourds and luffa), *Solanaceae* (eggplants, bell peppers), *Fabaceae* (peas, broad beans), and *Malvaceae* (okra). These are herbaceous and annual species of subtropical or tropical origin, contributing with fibre, vitamins and other non-nutritive but beneficial additions to the human diet.

Thus, eggplant fruit is rich in polyphenols, including hydroxycinnamic acid and its derivative chlorogenic acid, which have a potent antioxidant capability [1]. Eggplants and other immature fruits are also excellent sources of natural pigments and other antioxidant constituents such as chlorophyll a, chlorophyll b, and ascorbate [2].

Cucumbers have a very high percentage of water and very few calories, along with potential antidiabetic, hypolipidemic and antioxidant activity, mainly based on their high polyphenol content [3], up to 10 mg/100 g of flavonols and nearly 60 mg/100 g of proanthocyanindins [4]. Moreover, cucumber cucurbitacins exhibit anti-cancer activity as well as having purgative, anti-inflammatory and cosmetic pharmacological applications [5]. These medicinal properties are highly valued, both for their therapeutic activity within Indian and Chinese folk medicine and for use in the cosmetic industry [6].

In zucchini and other types of summer and gourd squash, the content of antioxidant compounds is of increasing interest for breeders, who have tried to increase the content of carotenoids and other nutritional compound and to improve external appearance and organoleptic characteristics [7,8].

Bitter gourd is notable for its medicinal properties. It is traditionally used in India, Korea, China and other Asiatic countries for the treatment of diabetes, its most common traditional use, but it is also used for the treatment of several illness such as dysmenorrhea, rheumatism, psoriasis and other [9]. Its medicinal properties are based on its high content of phenolic and saponin compounds, which are associated with antioxidant activity [10,11], and other compounds such as cucurbitane-type triterpenoids, and cucurbitane-type triterpene glycoside and insulin-like peptide [12,13,14]. The immature fruit of bitter gourd contains a high vitamin C content and it is a good source of vitamin A and some minerals such as iron [10,11,15].

Fresh luffa fruits are used as vegetables, but their seeds are used in traditional medicine in China due to antipyretic and anthelmintic properties [16]. The constituents of luffa fruits show antioxidant activity, due to their high vitamin C content, plus carotenoids and phenolic compounds such as catechins, flavonoids and anthocyanins [17]. These compounds show preventive effects on several types of cardiovascular complaints and cancer [18]. Du et al. [17] revealed that luffa fruits are rich in phenolic substances with high antioxidant potential such as cinnamic acid derivates and the flavonoid glycosides. Moreover, the luffa fruit is a recognized antibacterial agent due to its high content in tannins [19].

### 1.2. Harvest Time/Optimal Developmental Stage

The quality and the postharvest life of immature fruits are conditioned both by the developmental stage of immature fruits and the choice of harvest time [20]. Several harvesting indices (HI), including fruit size and shape, colour, texture, glossiness, among others, have been developed to assess the harvest time of fruit and vegetables [21]. The mature fruit HI are related to maximum growth and full ripening, a process required for the fruits to acquire their optimal organoleptic properties [22]. In immature fruits, however, the key parameter of the HI is fruit size, which is mainly chosen on the basis of consumer and market demands.

Since vegetable fruits are green at the edible stage, before seeds are fully enlarged and hardened, they can be harvested at various stages of development [23]. Zucchini HI, for example, are mainly based on the size and colour required by the marked (with an average length of about 20 cm), and the fruit being harvested just before hardening and darkening of fruit peel occurs and before undesirable seeds start to develop [24]. Firmness, colour and external glossiness are also common parameters used to define the HI of eggplants, cucumbers or bell peppers [25].

Cucumbers are harvested at nearly full size, but always before the seeds become hardened, at the moment when jelly-like material fills the seed cavity. The quality of a cucumber is also based on fruit glossiness, firmness and dark green peel [26].

Eggplants are harvested before reaching full size; however, there is a wide range of ontogeny stages at which they could be marketed [3] and the quality is primarily based on globular or elongated shape, firmness and a dark purple peel. Okra pods, on the other hand, are harvested while they are still immature; that is, having accumulated enough mucilage, but before becoming fibrous. This generally occurs within two to six weeks after anthesis [27].

### 1.3. Postharvest Physiology

Despite their great commercial and cultural importance, vegetable fruits are very high perishable commodities. Their epidermis is not yet fully developed and they are harvested at a developmental phase in which storage compounds have not accrued. Their high respiration rates, associated with high rates of dehydration and metabolism, lead to a rapid spoilage during storage [28]. The short commercial life of these fruits is therefore conditioned by their developmental stage at harvest time and their particular physiology during their postharvest storage conditions. Moreover, these products, due their tropical or subtropical origin, are susceptible to chilling injury (CI) when stored at low but non-freezing temperatures.

Three main factors are known to control the postharvest physiology of immature fruits: the respiration rate, the production of ethylene, and weight loss associated with dehydration. The respiration rate of immature fruit is much higher than that of mature fruit. While tomatoes, melons and mangoes have respiration rates ranging from 16 to 46 mg CO_2_ kg^−1^·h^−1^ at 10 °C, cucumbers, summer squash and okra have rates ranging from 30 to 143 mg CO_2_ kg^−1^·h^−1^ at the same temperature [25]. Respiration can be controlled by several factors such as temperature and atmospheric composition.

The speed of metabolic processes is increased by a factor of two for every 10 °C; therefore, respiration rates can be slowed down by reducing the temperature of fruit, although vegetable fruits are not adaptable to long-term storage because they are susceptible to CI [29]. The optimal storage temperature for immature fruit oscillates between 10 and 13 °C, as CI are induced at temperatures below 8–9 °C, this limits its long-distance distribution and marketing. Moreover, most vegetable fruits are susceptible to dehydration and mechanical injury [30]. Since mechanical injury also affects the fruit respiration rate, correct postharvest management that includes not only the control of the temperature but also the avoidance of mechanical damage is necessary to maintain the quality of the fruit [31].

The plant hormone ethylene is also important in controlling fruit physiology during its postharvest conservation period. Since they are immature at harvest, the fruits in question behave as non-climacteric and produces therefore a low rate of ethylene at harvesting and during storage [32,33]. In mature fruit the most conspicuous response to ethylene is related to ripening, but in green vegetables and immature fruits exposure to low levels of ethylene causes yellowing of green tissues, thus reducing the postharvest life of the commodities.

There are several factors promoting ethylene production, including mechanical injury, decay, insect damage and some types of stress such as low or high temperature. Megías et al. [34] demonstrated that cold-induced ethylene in zucchini is not produced during the cold storage period but after transferring the fruit to 20 °C. Also, the production level depended upon both the duration of cold storage and the period of conditioning time at room temperature [34,35]. It is indeed remarkable that, even after cold storage, ethylene production is dependent upon the level of sensitivity of the fruit to cold. Thus, sensitive cultivars of zucchini presented higher ethylene production, while less sensitive ones showed a lower level [36,37].

This cold-induced ethylene production has also been observed in other immature fruits such as cucumbers, eggplants and other climacteric and non-climacteric fruits such as citrus, kiwis and pears, among others [38,39,40,41,42]. It is always associated with an upregulation of ethylene biosynthesis genes [37,43,44,45,46,47].

Dehydration is also a major cause of fruit wilting and softening, and negatively affects nutritional quality. The dermal system of fruits and vegetable (cuticle, stomata, epidermal cells, trichomes) is responsible for regulation of water loss. This system is developed during the growth and development stages of the fruit so that it can fulfil its function when the fruit is mature [48]. The partial development of the dermal system makes immature fruits highly susceptible to water loss. The first symptom associated with fruit dehydration is wilt, but in cucumbers it has been demonstrated that dehydration also upregulates the activity of the cell-wall-degrading enzymes like poligalacturonase and pectinesterase, which finally leads to fruit softening and shrivelling [49]. Moreover, water loss increases ethylene production, which, as Lurie et al. [50] and Kubo et al. [49] pointed out, could explain the accelerated senescence observed in bell peppers and cucumbers.

Massolo et al. [51] found that eggplant fruit treated with 1-methylcyclopropene (1-MCP) maintained fruit firmness concomitantly with a reduction in water loss, this probably being caused by a delay produced by 1-MCP in senescence of the calyx.

## 2. Chilling Injury and Oxidative Stress

### 2.1. Chilling Injury (CI)

The storage of fruit under cold conditions is a generalized technology used to avoid rapid decay and maintain quality. Low temperature slows down many of the processes responsible for the deterioration and loss of quality in vegetable fruits [52]. However, many fruits and vegetables, including immature fruits, are susceptible to chilling injury (CI). Their storage at cold but non-freezing temperatures triggers a number of CI symptoms that irreversibly reduce the external and internal quality of the product [53].

Chilling injury refers to a syndrome that involves several physiological events, as well as the characteristic and recognizable symptoms of cold-stored fruit. The type and extent of this syndrome varies with the species, cultivar, cold storage conditions and other factors, including farming conditions. For example, while in eggplants a characteristic expression of chilling injury includes a net browning of the pulp [51], in cucumbers and zucchinis the damage may include surface pitting, dehydration and large sunken areas, as well as discoloration. There is, therefore, no one single symptom or type of damage that is common to all products and would allow for the assessment of CI in all commodities.

Nevertheless, there are instances of several symptoms appearing together that can be recognized as the consequence of cold storage. Some CI changes occur at cellular level, including alterations of membrane structure, cell plasmolysis and increased electrolyte leakage [54,55]. Others changes imply alterations in the metabolism, including higher levels of ethylene production, and the accumulation of abnormal compounds such as malonyldialdehyde (MDA) as a resulted of either anaerobic respiration or oxidative damage [24,37].

One of the most notable CI-associated alterations, however, affects the external appearance of the fruit and include damage to the fruit surface, including pitting, large sunken areas, discoloration, translucent water-soaked spots and water-soaked areas and deep lesions that can reach the subepidermal tissues [55]. These macroscopic changes are commonly used to assess the extent of cold damage [24,36,46,56].

The most common CI symptoms in immature fruit include weight and firmness loss, electrolyte leakage, and appreciable damages to the fruit surface. In cucumbers, watery pitting is the main symptom of CI [57]. Ultrastructural analysis of this damage has revealed that they are associated with cracks in the cuticle and the sinking of epidermal cells near the stomata, which together lead to a raised transpiration rate, as well as a decay due to the growth of necrotrophic fungi [55,58]. Another specific symptom of CI in cucumbers is discoloration due to chlorophyll degradation that takes place at low temperature [59].

In the case of zucchini, pitting is the main chilling symptom. Microscopical analysis of this pitting has shown that the surface depressions are caused by cell death and cell collapse, associated with solubilisation of pectin and cell wall degradation [60]. When the symptom of pitting is visible, chilling decay is irreversible; Balandrán-Quintana et al. [61] suggested that respiration rate may be an indicator of chilling damage before visible symptoms appear and injury becomes irreversible.

At the mature green or breaker stage, before the fruit has completely ripened, bell peppers are also susceptible to cold storage, although the degree of susceptibility depends on the cultivar [62,63]. Green bell peppers show similar symptoms to those described as appearing in cucumbers and zucchini, along with shrivelling, resulting from fruit moisture loss and also seed browning [62,64]. These are also major chilling symptoms in eggplants [65,66]. Browning is caused by oxidation of phenolic compounds by the enzyme polyphenol oxidase (PPO), which seems to be dependent on low temperature. This enzyme catalyses the o-hydroxylation of monophenols to *o*-diphenol and further oxidation of *o*-diphenols to *o*-quinones, which react with amino acids or proteins, generating brown pigmentation [67,68].

Okra is moderately susceptible to CI, with the most common symptoms of chilling injury being water-soaked lesions, pitting, discoloration, appearance of mould or mildew and increased decay (especially after removal to warmer temperatures, as during marketing). The sensitivity to CI varies according to okra cultivars and phenological stage of pods. Young mucilaginous pods are more sensitive than larger pods. Usually the symptoms appear after two days of cold storage if the temperature is as low as 2 °C. The green pods turn a brownish olive-green, while yellow cultivars turn brown or brownish-red [69,70,71].

Luffa fruits are highly perishable and their postharvest life is no more than one week at room temperature. During the process of decay, the fruits appear yellow and are wilting. Luffa is highly sensitive to chilling injury and the most visible symptoms include discoloration, watery brown or black spots on and under the epidermis, and a higher level of decay including severe decomposition, especially if the fruit is being rewarmed after cold storage [72,73]. Table 1 shows the chilling injury symptoms in some vegetable-like fruits.

### 2.2. Oxidative Stress

#### 2.2.1. Overview

The environmental and physical changes that the produce undergoes before and after harvest may induce the production of reactive oxygen species (ROS), which are the cause of oxidative damage during the postharvest of immature fruit and vegetables, inducing decay of the product and loss of quality. Oxidative stress starts with an elevated production of ROS as a result of numerous processes such as photosynthesis, respiration, photorespiration and oxidative burst, which occur at different cellular locations and within different cellular organelles [77,78]. ROS include superoxide anion, hydrogen peroxide, hydroxyl radical, nitric oxide, and peroxynitrite. If the production of ROS increases dramatically, as occurs under environmental stress, hydroxyl radical reacts with membrane lipids, inducing peroxidation, which leads to membrane degradation. Malondialdehyde (MDA) is a product of this lipid peroxidation, and is used as an indicator of stress in some tissues [79].

During immature fruit postharvest, oxidative stress avoidance is important for the maintenance of fruit quality. Two strategies have been developed to avoid or tolerate oxidative stress, generating a response that includes metabolic changes at biochemical and molecular level [80]. These changes include the induction of the enzymatic and non-enzymatic mechanisms of the antioxidant defence: superoxide anions are detoxified by the enzyme superoxide dismutase (SOD), which produces hydrogen peroxide that can be scavenged by catalase (CAT) and several peroxidases (POD) such as thioredoxin peroxidase and glutathione peroxidase, as well as the enzymes belonging to the Foyer–Halliwell–Asada cycle [78]. In this cycle, ascorbate peroxidase (APX) uses ascorbate for the reduction of hydrogen peroxide. The oxidized ascorbate is recycled by monodehydroascorbate reductase (MDHAR) and dehydroascorbate reductase (DHAR), in this case oxidizing glutathione that is reduced by glutathione reductase (GR) [81,82]. As for non-enzymatic antioxidants, there are several metabolites in plants such as carotenoids, phenolic compounds, ascorbate and glutathione that can be used as ROS scavengers. In tissues with a high rate of respiration such as developing fruit, in addition to ROS scavenging mechanisms the mitochondrial alternative oxidase (AOX) provides an alternative electron flow. This activity can reduce the production of ROS in situations where the electron transport chain is saturated or suffers some damage [83].

Another way to prevent oxidative stress is the activation of metabolic pathways involved in the maintenance of redox status, such as the GABA shunt pathway [84]. In *Arabidopsis*, mutants disrupted in the enzyme of the GABA shunt succinic semialdehyde dehydrogenase were unable to scavenge hydrogen peroxide [85].

When the generation of ROS exceeds the capacity of the plant tissue to maintain cellular redox homeostasis, oxidative stress appears [86]. This stress in fruit and vegetables can be detected either directly as accumulation of ROS, MDA, and the apparition of brown pigments added to enhanced electrolyte leakage, or indirectly as changes in enzymatic and non-enzymatic antioxidant systems [87]. With regard to acclimatisation to abiotic stress, several phytohormones play a main role in the activation of the response. It has been reported that an intricate interplay between phytohormones and ROS [88], added to elevated levels of ROS, also modulate the levels and functions of phytohormones [80].

#### 2.2.2. Low Temperature and Oxidative Stress in Vegetable Fruit

The storage of vegetable fruit at low temperature is necessary in order to reduce fruit respiration, thus avoiding postharvest losses. However, cold storage generates CI in many vegetable fruits, such as cucumbers, zucchini and eggplants, by inducing ROS production [3,36,89]. It was proposed that lipid peroxidation was associated with CI, after the study by Parkin and Kuo [79] in which chilling-induced lipid degradation in cucumber fruit was detected. The plastids were suggested as the site of chilling-induced peroxidation. By monitoring electrolyte leakage and changes in thylakoid lipids in cold-stored cucumber fruit, Hariyadi and Parkin [90] proved the existence of oxidative stress during cold storage, and confirmed the sensitivity of plastid membranes to chilling.

Fruit are structures with higher respiratory activity; however, low-temperature storage may induce an uncoupling in the respiratory chain, giving rise to ROS. In plants exposed to low temperature a sharp increase in their cyanide-sensitive respiration has been reported, mainly due to an induction of AOX activity [83]. AOX is a very efficient way of diverting the electron flow and reducing the generation of superoxide anion, hydrogen peroxide, and hydroxyl radical. Low temperature also reduces antioxidant enzymatic activity in fruit of chilling sensitive cultivars, thereby decreasing their ability to cope with elevated ROS production [36,62]. In addition, treatments used to reduce the damage caused by cold storage in sensitive fruits lead to a reduction of oxidative stress by increasing the antioxidant defence [24,91,92,93,94].

In cucumbers, a decrease of the non-enzymatic antioxidant glutathione, α-tocopherol, and ascorbate has been found during cold storage [90]. More recently, ROS accumulation has been assessed in cold-stored cucumber fruit, detecting a gradual increase in hydrogen peroxide content and the rate of superoxide anion production during storage [89,95].

In this species, the chilling tolerance depends on the cultivar [59] and is affected by fruit maturity. Fruits at earlier developmental stages are more susceptible to chilling stress, associated with a higher MDA content and electrolyte leakage, than more mature fruit [96,97]. At earlier developmental stages, cold-stored cucumber fruit shows a lower transcription levels of the main antioxidant enzymes (SOD, CAT, POD, MDHAR, DHAR, GR) even after rewarming, which may be the cause higher oxidative damage [97]. At later stages of development, however, the fruit displays a higher ascorbate content, which improves antioxidant capacity and allows longer cold storage [96].

Many studies have been undertaken to determine the defence mechanisms of the zucchini fruit against CI [37,46,60,94,98,99,100,101]. Whilst analysing the postharvest condition of several cultivars of zucchini fruit, it has been demonstrated that chilling tolerance in this species is also cultivar-dependent [35,36]. Cold storage induces the accumulation of hydrogen peroxide in both exocarp and mesocarp tissues of this fruit [102]. In addition, a strong positive correlation has been detected in exocarp tissue between hydrogen peroxide and MDA content and CI, whereas CAT activity and CI symptom correlate negatively in this same tissue [36].

Soluble sugars also play an important role in the ROS balance under conditions of stress, either by acting as ROS scavengers, as osmoprotectants or by stabilizing cellular membranes [103,104]. The fruit of a chilling-tolerant cultivar of zucchini accumulated higher levels of soluble sugars under cold stress, associated with an increased antioxidant defence [36,99]. Moreover, the induction of the GABA shunt pathway in cold-stored zucchini fruit has been considered as a contribution to the acquisition of chilling tolerance by increasing the production of reducing agents and energy providers [91,92,100]. It can therefore be deduced that an enhanced antioxidant defence and energy status improves the quality and cold tolerance of zucchini fruit.

In green bell peppers, it has been reported that low-temperature storage causes a decrease in SOD and CAT activities in sensitive cultivars at the most susceptible stages of ripeness [62]. This CAT response could be related to the ultrastructure changes that take place as a consequence of chilling damage, such as plastid degradation and the disappearance of peroxisomes [105]. In this study, a proteomic analysis proved that one of the main processes affected by chilling in pepper fruit is redox metabolism. Among the antioxidant responses in peppers was a significant increase in the induction of AOX in cold-stored fruit [106]. Specifically, this work reported the accumulation of two AOX, one of them (*CaAOX1*) being an unprocessed transcript due to altered RNA splicing that takes place at 0 °C. Although *CaAOX1* is expressed, it is accumulated both during cold storage and during treatments that improve chilling response, induce earlier and higher expression levels of this gene. Another chilling symptom in bell peppers is seed browning, which has also been also associated with certain stages of maturity of this fruit [64]. These authors have reported lower of levels of CAT and POD but higher Superoxide Dismutase (SOD), Lipoxygenase (LOX), Phenylalanine Ammonia Lyase (PAL), Polyphenol Oxidase (PPO) activity in seeds during cold-sensitive stage, as well as increased MDA and saturated fatty acids content.

Eggplants are affected by CI when stored at temperatures below 10 °C, but their sensitivity differs among cultivars, and depends on: the size at harvest; the developmental stage and environmental conditions. However, the differences in chilling susceptibility within the normal eggplant harvest stages are not clear yet. Zaro et al. [3] showed, in two cultivars, that during the early stage of development (baby eggplant) the chilling damage was less than in later stages; these latter containing a higher content in antioxidants. In the darker cultivars of eggplant, the epidermis is rich in anthocyanins, such as delphinidin-3-(p-coumaroyl rutoside)-5 glycoside (nasunin) and/or delphinidin 3-rutoside (D3R), both of which contribute to hydroxyl radical and superoxide anion radical scavenging activities [107]. However, these darker cultivars are very susceptible to browning when stored for prolonged periods or at low temperatures. Indeed, the levels of phenols have been positively correlated with the incidence of browning [108]. Concellon et al. [66] reported an increase in membrane and organelle disruption during the cold storage of eggplants. This loss of cell compartmentalization makes phenols accessible to PPO. In a similar way, calyx browning correlated positively with PPO activity [109].

## 3. Postharvest Treatments that Reduce Oxidative Stress and Chilling Injury

Different postharvest technologies have been developed to avoid or delay the development of chilling injury during the postharvest storage of immature fruits. The current market trend is to avoid synthetic chemicals, whilst encouraging the use of sustainable postharvest technologies based on physical treatment (temperature, relative humidity and modified atmosphere packaging) and biochemical treatments using natural products and growth regulators (Table 2).

### 3.1. Physical Approaches

#### 3.1.1. Temperature Treatments

Postharvest heat treatments prior to low-temperature storage have been used to prevent or mitigate the action of CI in certain immature fruits, including cucumbers [110,111], peppers [112] and zucchini [46,60].

Hot water dipping (HWD), intermittent warming (IW) or hot water rinsing and brushing (HWRB) have prolonged the shelf life of vegetables and fruits [113]. These short-term bathing techniques, along with intermittent warming (IW), induce heat shock protein (HSP) genes [113,114], polyamines (PA) production [93,112] and the natural antioxidant defence system within the fruit itself [115,116]. This maintains the quality of some immature fruit commodities [45,117,118,119,120]. A recent report in zucchini has also shown that warm-water treatments are effective for maintaining fruit firmness while preventing the proliferation of fruit microflora [121].

Postharvest preconditioning treatments at moderate temperatures before cold storage allow a progressive adaptation of the fruit to chilling temperatures. In zucchini, it has been reported that temperature-preconditioning treatments at 15 °C for two days, before storage at the chilling temperature of 4 °C, reduces CI symptoms by preventing the deterioration of the cell membrane and improving the antioxidant status of the preconditioned fruit [60,122,123].

These treatments were able to reduce lipid peroxidation and ion leakage, which are indicative of a lack of membrane integrity, while diminishing H_2_O_2_ content, thereby inducing the activities of antioxidant enzymes such as ascorbate peroxidase (APX) and catalase (CAT), and increasing ATP pool and proline accumulation [60]. The reduction of pitting and fruit weight in temperature-preconditioning zucchini fruits was also accompanied by a reduction of both cold-induced ethylene and the fruit respiration rate in the more chilling-susceptible cultivars [46], suggesting that ethylene is also a modulator of CI in this immature fruit.

In the case of cucumbers, a preconditioning treatment at 10 °C encouraged chilling tolerance associated with a reduced accumulation of MDA and ROS and with induced activity of SOD, APX, and CAT, together with a higher content of the ROS scavengers ascorbic acid and glutathione [124].

#### 3.1.2. The Use of Controlled and Modified Atmospheres

Fruit respiration rate and fruit dehydration are two physiological aspects that limit the shelf life of fresh fruits during their postharvest storage period [125,126,127]. The use of controlled atmosphere (CA), modified atmosphere packaging (MAP), and edible coatings alters the gaseous atmosphere where the main active gases (O_2_, CO_2_, ethylene and H_2_O vapour) surrounding the fruit [128,129,130,131,132,133]. Thus, fruit quality was better maintained under CA and MAP, this being associated with reduced oxidative stress in the refrigerated immature fruits of different species such as cucumbers, peppers and zucchini squash.

Although rarely used during storage of immature fruit commodities, there are some reports demonstrating the positive effects of CA on cucumbers and zucchini [94,134,135,136]. Fahmy and Nakano [134] reported that cucumber fruit stored under CA with low O_2_ reduced weight loss, changes in skin colour, electrolyte leakage and MDA accumulation. Low O_2_ storage atmosphere were also responsible for reducing CI in zucchini squash, maintaining higher levels of the polyamines spermidine and spermine [137]. Exposure to 40% CO_2_ prior to chilling storage at 2 °C were found to be effective in reducing CI in zucchini squash, while reducing abscisic acid, putrescine, spermidine and spermine content [136]. Under CA with a high level of O_2_, however, zucchini fruit slightly induced AOX, as well as SOD, APX and CAT antioxidant activity together with total phenolics, suggesting the involvement of alternative respiratory pathway during chilling stress [94].

#### 3.1.3. The Effects of Different Types of Wrapping

The use of plastic coverings with differing levels of permeability to active gases produces a balance in CO_2_ production and O_2_ consumption that passively modifies the atmosphere of freshly-packaged produce. Due to its comparatively low cost, MAP has been widely used in the industry for the maintenance of fruit quality and the extension of product shelf life, in both mature and immature fruits [53,75,119,128,138,139,140].

Individual shrink wrapping (ISW) is a form of MAP that prolongs the shelf life and maintains the harvest freshness of fruits and vegetables. The main advantages of ISW include a reduction in CI, together with reductions in fruit dehydration, deformation and decay, which prevent secondary infection. In cucumbers and peppers, ISW using polyethylene film has been proved to be useful for extending fruit shelf life and reducing weight loss while retaining freshness, colour and firmness [135,141,142,143].

Shrink packaging in combination with HWRB treatment reduced weight loss, softening, decay incidence and CI in green peppers, while reducing LOX activity and electrolyte leakage [114,118]. Shrink-wrap film was also used to extend the shelf life of zucchini stored at 4 °C, reducing the loss of both fruit weight and firmness as well as reducing CI symptoms. This coincided with a reduction of cold-induced ethylene production, a downregulation of ethylene biosynthesis and signalling genes, a decrease in fruit respiration rate and the accumulation of oxidative stress metabolites such as H_2_O_2_ and MDA [24].

#### 3.1.4. The Effects of Edible Coatings

Edible coatings also preserve the quality of vegetable fruits by reducing water loss and CI symptoms during postharvest storage, and by activating the antioxidant system that reduces membrane damage and prevents flesh browning. Alone or in combination with essential oils from diverse origins, edible coatings are being increasingly used for their non-toxic, biodegradable and biocompatible properties, reducing thereby the need for disposable non-degradable packaging materials. The application of ceramide lessens CI symptoms in pepper fruits stored at 4 °C, delays the degradation of chlorophylls and soluble proteins, suppresses the accumulation of MDA by maintaining the integrity of the cell membrane, and enhances the activity of the antioxidant enzymes POD, CAT, and APX [144]. Chitosan-based coatings were also observed to improve the shelf life of cucumbers [145,146], zucchini [121], bell peppers, [147,148,149,150] eggplants [151] and sponge gourds [152].

### 3.2. Chemical Approaches

Given that oxidative stress and CI symptoms are controlled by growth regulators and metabolites inducing signal transduction mechanisms, many postharvest technologies are based on treatments using hormones, hormone inhibitors, or metabolites involved in stress responses. These include ethylene inhibitors, brassinosteroids, abscisic acid (ABA), salicylic acid, jasmonic acid and polyamines. Others are based on natural non-toxic products.

#### 3.2.1. Ethylene (ET)

ET regulates a wide range of biochemical, physiological and developmental processes that can damage climacteric and non-climacteric fruits during postharvest storage [153,154,155]. The use of ET biosynthesis and response inhibitors has been extensively implemented in the industry to extend postharvest self life and to delay senescence and thereby decay of the fruit.

The gaseous ET analogue 1-methylcyclopropene (1-MCP) can irreversibly bind to ethylene receptors, thus avoiding subsequent ethylene response [156,157]. Postharvest treatments with 1-MCP are effective, stable over time and non-toxic, but their effectiveness is highly variable and depends upon the species and cultivar employed, the treatment conditions and the stage of ripening of the fruit. It has been demonstrated that 1-MCP successfully prevents deterioration of fruit quality by delaying ripening or senescence in many fruits, but also by inducing chilling tolerance. This has been reported in the immature fruits of eggplant, peppers and zucchini [37,51,158].

In zucchini, 1-MCP delays the onset of CI symptoms such as pitting and fruit weight loss in the fruit of the most chilling-susceptible cultivars, which coincides with a reduction in the respiration rate and cold-induced ET and includes a downregulation of ET biosynthesis and signalling genes [37]. These results indicate that ethylene is not only a response of the fruit to cold damage, as occurs upon rewarming, but could also play a regulatory role in the onset of CI in the non-climacteric zucchini fruit.

Postharvest technologies based on ethylene removal, such as that of catalyst titanium dioxide (TiO_2_), and the ethylene absorbents CaCl_2_ and KMnO_4_, have been employed to prolong the shelf life of fruit produce [159]. A new palladium-based ethylene adsorbing technology has also been developed, which scavenges ethylene for the control of fruit ripening [159]. Nevertheless, none of these treatments have been so far used for the postharvest management of immature fruits.

#### 3.2.2. Brassinosteroids (BRs)

BRs have also been reported to be effective in delaying CI symptoms in immature fruits. Wang et al. [160] showed that BRs mitigate the action of CI in green bell peppers, accompanied by a reduction in electrolyte leakage, a reduction MDA content, and enhanced the activity of antioxidant enzymes such as CAT, APX, and GR. In the same way, Gao et al. [161] have found that BR alleviated CI and flesh browning in eggplant fruit maintaining membrane integrity and moisture content, by declining in the accumulation of H_2_O_2_ and by inducing total phenolics and PAL, PPO, and POD activities.

#### 3.2.3. Abscisic Acid (ABA)

ABA is accumulated in plants in response to many stress factors, including drought, salinity and cold [162,163]. The accumulation of ABA prevents cellular dehydration by promoting stomata closure and the accumulation of osmoprotectant molecules such as soluble sugars and proline [164,165]. Moreover, ABA can induce the antioxidant defence response which reduces the effects of the oxidative stress that appear during dehydration [166], and can promote the induction of genes that are involved in cold and dehydration tolerance. These genes are known as *COR* (Cold Regulated) and ABRE (ABA Responsive Element Binding) genes [167,168,169].

In vegetable fruits, we have recently tested that the application of ABA induces cold tolerance in the chilling-sensitive zucchini cultivar Sinatra, while treatment with the ABA inhibitor sodium tungstate reduces the chilling tolerance of the chilling-tolerant cultivar Natura (Carvajal et al., unpublished).

#### 3.2.4. Salicylic Acid (SA) and Jasmonic Acid (JA)

SA and JA, as well as their methyl esters, known as methyl salicylate (MeSA) and methyl jamonate (MeJA), are natural and safe signalling molecules that were applied to fruits and vegetables to enhance CI tolerance and to maintain the postharvest quality of fresh produce [170]. In luffa fruits (sponge gourd), the application of SA alleviates postharvest CI by decreasing electrolyte leakage, reducing the accumulation of MDA and total phenolics, enhancing the activity of antioxidant enzymes such as SOD, CAT and APX, and inhibiting the activity of phenylalanine ammonia-lyase (PAL) and polyphenol oxidase (PPO) [73]. In order to facilitate salicylic acid biological function, efforts have been made to synthesize salicylic-acid-grafted chitosan. The so called salicyloyl chitosan was utilized because of its superior water solubility and adhesiveness to fruit surface [171]. Zhang et al. [116] reported that the salicyloyl chitosan coating alleviated chilling injury and weight loss in cucumber fruits, which was associated with reduced respiration rate, lower electrolyte leakage, reduced MDA accumulation, and higher activity of the antioxidant enzymes SOD, CAT, APX and GR.

JA and MeJA are also regulators of stress responses [172,173]. The induction of JA genes in *Arabidopsis thaliana* [174], and rice [175] conferred enhanced chilling tolerance. In species with immature fruits, it has been reported that MeJA treatment alleviates CI and reduces ethylene production in cold-stored eggplant fruits [176], but was ineffective in reducing CI in zucchini squash [177]. In green bell peppers, MeSA and MeJa enhance CI tolerance by inducing the expression of the *AOX* gene, thereby maintaining the balance between ROS production and general antioxidant system activity [178]. In cucumbers, MeJA induces chilling tolerance by inhibiting H_2_O_2_ generation, and enhancing both CAT activity and CAT gene expression [179].

#### 3.2.5. Polyamines (PAs)

PAs are a group of phytohormone-like aliphatic amine compounds, major types of which are the triamine spermidine (Spd), the tetramine spermine (Spm), and their diamine precursor, putrescine (Put). They produce pleiotropic effects in plants, including defence against various abiotic stresses. Since PA and ET share the same precursor, S-adenosylmethionine (SAM), high levels of PA would provoke a reduction in ET levels followed by an increase in CI tolerance as observed in peppers [110,112] and in zucchini [98,99,122,180].

In zucchini, the enhanced tolerance to CI in putrescine-treated fruits was associated with reduced levels of hydrogen peroxide and malondialdehyde, which could be related to changes in LOX activity [180]. These advantageous changes appear to occur by activating the antioxidant system via the accumulation of ascorbate and ferric reducing/antioxidant power (FRAP), and by inducing the activities of APX, CAT and GR antioxidant enzymes [92]. The response of cold stored zucchini fruit to PA treatments could be also due to other physiological responses that protect cells from oxidative damage, including γ-aminobutyric acid (GABA) degradation via the GABA shunt pathway [180] and via the accumulation of soluble sugars including glucose, fructose and raffinose, as has been observed in putrescine-treated zucchini fruits under cold storage [92,99].

PAs also enhance the chilling tolerance of cucumber plants through modulating antioxidative system in the leaf [181]. There is, however, no data concerning the effect of these plant growth regulators on the oxidative status and CI tolerance of the cucumber fruit. 

#### 3.2.6. Nitric Oxide (NO)

Nitric oxide (NO) is an important signalling molecule involved in many plant physiological processes [182]. It has been reported that NO protects plant cells against oxidative stress by reducing ROS accumulation, and has been used as a postharvest treatment for maintaining fruit quality and alleviating CI symptoms. In cucumbers, the treatment with NO was effective in alleviating CI symptoms, while reducing oxidative stress damage due to a decrease in O_2_^−^ and H_2_O_2_ and by activating the antioxidant enzymes CAT, SOD, APX and POD [95,179]. NO fumigation was also applied to extend the postharvest life of green beans [183].

#### 3.2.7. Other Chemical Treatments

Calcium treatments have been shown to retain the firmness and quality of vegetables and fruit as Ca contributes to the structural integrity of plant cell walls and membranes [184]. De Bruin et al. [121] reported that in zucchini fruit treated with CaCl_2_ flesh firmness was effectively maintained during 14 days of storage, in contrast to the control fruit, which showed a decline in flesh firmness after the first seven days of storage.

6-Benzylaminopurine (6-BA) is closely related to plant stress tolerance. Little research has been carried out on the postharvest cold tolerance of vegetable fruits. Chen and Yang [89] have shown that 6-BA alleviated CI in cucumber fruit through improving antioxidant enzyme activity and total antioxidant capacity, and by maintaining higher levels of ATP content and energy charge. Fruits treated with 6-BA maintained higher levels of chlorophyll, ascorbic acid, total phenolics and total antioxidant capacity. Furthermore, under chilling stress, this treatment reduced the increase in membrane permeability and lipid peroxidation, delayed the increase in both O_2_^−^ H_2_O_2_ production, and increased the activities of SOD, CAT, APX and GR.

Massolo et al. [185] have stated the effect of postharvest cytokinin (CK) treatment on refrigerated, round, soft-rind squash. CK-treated fruit showed slower deterioration and dehydration, and remained firmer than the control. The treatments prevented phenolic-compound accumulation and decreased pectin solubility. The treated squash had higher levels of tightly-bound polyuronides, indicating a substantial delay in cell wall dismantling. CK sprays also reduced neutral sugar solubility from pectin-rich fractions [185].

Glycine betaine (GB), a stress-ameliorating compound, has been used to reduce CI in sweet peppers [186]. The authors suggested that GB increases chilling tolerance by inducing the activity and expression of POD, CAT APX and GR antioxidative enzymes and genes, thus alleviating the potential injury resulting from CI. The amelioration of CI in sweet peppers by GB was associated with a reduction in electrolyte leakage, MDA content, and lipid peroxidation.

### 3.3. Breeding and Biotechnological Approaches

Plant breeding and biotechnology approaches are strategies that show potential for decreasing oxidative stress while enhancing CI tolerance in fruits. Numerous examples of transgenic plants showing reduced CI are described in the literature, although a few of them refer to the postharvest quality of immature fruits.

Breeding for tolerance to postharvest CI has rarely been used for enhancing the postharvest quality of immature fruits. The observed genetic variability of CI among commercial zucchini hybrids [36], and the greater variation among traditional local varieties in gene banks [35], opens up the possibility to achieve new zucchini cultivars by combining different sources of CI tolerance in zucchini.

With this objective in mind, Megías et al. [35] have recently described a study of the mode of inheritance of a source of cold tolerance in zucchini, finding a cosegregation between cold-induced ethylene production and CI index and weight loss, suggesting that cold-induced ethylene can be used as a marker to select postharvest cold tolerance in segregating populations.

Several biotechnological approaches have been developed to induce tolerance to oxidative stress by enhancing enzymatic and non-enzymatic mechanisms of ROS detoxification. The accumulation of some antioxidant compounds including ascorbate, glutathione, tocopherols and tocotrienols, polyphenols, carotenoids, flavonoids and anthocyanins protect plants against oxidative stress, but the most used ROS scavenging mechanisms in transgenic plants were those that overexpress genes encoding for the enzymes involves in O_2_^−^ and H_2_O_2_ detoxification: SOD, PX and CAT (Table 3). In recent years, post-transcriptional mechanisms based on non-coding regulatory micro RNA (miRNA) have been also used to induce tolerance towards multiple stresses, including oxidative and abiotic stresses [189]. Most of these biotechnological approaches have been developed and tested in model species, but some of them were also tested and shown to be effective in immature fruit species (Table 2).

Lee et al. [190] reported the generation of a transgenic cucumber plant that overexpress a gene from cassava (*mSOD1*) under a fruit specific promoter, which was used to produce an anti-ageing cosmetic from the cucumber fruit. Similarly, the overexpression of the tomato *Cu*/*Zn SOD* gene in peppers increased regeneration efficiency while reducing H_2_O_2_ accumulation [191]. The overexpression of *APX* was a means of inducing tolerance to oxidative stress in cucumbers [192] and bell peppers [193], while *APX* genes from eggplants, peppers and sponge gourds were found to be effective in conferring flooding and drought tolerance and in reducing oxidative injury in *Arabidopsis* and rice [193,194,195]. The cucumber *NITRIC OXIDE ASSOCIATED 1* gene (*CsNOA1*) is induced by chilling stress, and its constitutive overexpression in cucumbers led to a greater accumulation of soluble sugars and starch, plus an upregulation of *Cold-regulatory C-repeat binding factor 3* (*CBF3*) expression, and a lower CI index in cucumber seedlings [179]. The overexpression of other genes, including pepper *LOX* and *S-ADENOSYLMETHIONINE DECARBOXYLASE* (*SAMDC*) genes, reduced lipid peroxidation in membrane cells and reduced ROS, meanwhile inducing higher tolerance to *Arabidopsis* biotic and abiotic stresses [196,197,198].

Transcription factors and upstream regulators involved in abiotic and oxidative stress responses have been manipulated in transgenic plants with immature fruits. The *Arabidopsis CBF1* gene driven by the inducible promoter *RD29A* enhanced chilling stress tolerance in cucumbers, concomitantly with an increase in activity of antioxidant enzymes SOD and CAT and a higher free proline content and relative water content in leaves [199]. In the same way, transgenic cucumbers overexpressing the *ICE1* gene are more tolerant to chilling stress, showing enhanced expression of cold-responsive genes, a greater concentration of sugar and proline, and a reduced accumulation of MDA [200]. In eggplants, the overexpression of the *R2R3 MYB* transcription factor gene *SmMYB1* resulted in transgenic eggplants that accumulate higher concentrations of anthocyanin in leaves, petals, stamens, and also in the fruit peel and flesh. The GM seedlings exhibited a higher antioxidant activity concomitant with a greater tolerance to freezing and better recovery under rewarming conditions [201]. The ectopic expression of pepper *RECEPTOR-LIKE KINASE 1* (*CaRLK1*) and ankyrin repeat domain zinc finger transcription factor gene *CaKR1* demonstrated that they function, respectively, as negative and positive regulators of ROS accumulation, resulting in plants that were respectively more sensitive and tolerant to oxidative stress [202,203].

Most of the biotechnological studies detailed above have focused on the vegetative organs of plants, few having been conducted on the fruit. Future studies are needed, therefore, so as to address the effects of these antioxidant transgenes on ROS scavenging systems and chilling tolerance in the immature fruit of marketable species.

## 4. Conclusions and Perspectives

Immature vegetable-like fruits are highly perishable commodities. They are in a phase of development in which the metabolism, and therefore respiration rate and transpiration, is very high. Although cold storage decreases their metabolism, these fruits are very susceptible to CI, which is associated with an increase in the production of ROS and the induction of oxidative stress. To avoid such stress, a series of coordinated protective mechanisms are triggered in the fruit, including enzymatic and non-enzymatic antioxidant defence mechanisms, the activation of an alternative electron transport by mitochondrial Alternative Oxidase, and the induction of metabolic pathways that maintain the redox balance.

Several physical, chemical and biotechnological approaches have been developed in immature fruit to reduce post-harvest losses caused by cold damage, many of which are aimed at reducing ROS production and avoiding oxidative stress. In addition to their effectiveness in preventing CI symptoms, new postharvest technologies should be increasingly sustainable, promoting the use of natural products and avoiding chemical compounds that may affect food safety and environmental preservation. In this sense, plant breeding is offered as a sustainable solution that should be taken more and more into account for the development of new varieties that are increasingly tolerant to oxidative stress and CI. Moreover, the biotechnological strategies should not only be used to improve our understanding of oxidative stress regulation, but also to provide tolerant plant material for future breeding programs.

## Figures and Tables

**Table 1:** Chilling injury symptoms in some immature fruits.

Immature Fruit	Symptoms	Threshold Temperature (°C)	References
Cucumber	Surface pitting, increased yellowing and disease susceptibility, water-soaked areas of the flesh	10–12	[26,38,55]
Eggplant	Surface pitting and scald, browning of the flesh and seeds	8–12	[51]
Bell pepper	Surface pitting, water-soaked areas, seed browning, and decay		[62,74]
Okra	Discoloration; water-soaked areas; surface pitting; exuding lesions, and decay by mould or mildew, calyx discoloration	7–10	[27,75]
Zucchini	Surface pitting, large sunken areas, dehydration, discoloration	7–10	[24,36]
Bitter gourd	Pitting that coalesced to form large sunken dark brown pits; surface discoloration; internal tissue breakdown; decay	8–10	[76]

**Table 2:** Postharvest treatments alleviating oxidative stress and CI in immature fruits.

Postharvest Technologies	Technology	Species	Effects	References
Physical	Heat treatment	Cucumber	Reduced electrolyte leakage, chilling-induced ethylene, and ACS and ACO activity	[117]
			Reduced electrolyte leakage and MDA, and enhanced PLD and LOX activity	[110,111]
		Green bell pepper	Reduced CI, electrolyte leakage and LOX activity	[114]
			Reduced weight loss, softening, decay and CI	[118]
			Enhanced PA content and increased PAL and PPO activity	[112]
			Reduced CI, maintained firmness, and delayed unsaturated fatty acid accumulation	[187]
		Zucchini	Induced *HSP* genes, maintained flesh firmness	[113]
		Eggplant	Retarded CI, reduced spermidine	[119]
	Temperature preconditioning treatment	Zucchini	Alleviated CI and weight loss, reducedH_2_O_2_, MDA and ascorbic acid content, and induced activity of antioxidant enzymes	[46,91,93]
		Cucumber	Increased soluble solids, ascorbic acid, and MDA, O_2_^−^ and H_2_O_2_, induced activity of antioxidant enzymes, and the scavengers AsA and glutathione	[124]
	Controlled atmospheres. CO_2_ and O_2_ treatments	Cucumber	Alleviated chilling injury, weight loss and changed in peel colour, maintenance of electrolyte leakage and MDA	[134]
		Zucchini	Reduced CI, increased levels of spermidine, spermine and total phenolics, induced activities of alternative oxidase, SOD, APX and CAT	[94,136,137]
	Controlled atmospheres. Use of plastic covers	Green bell pepper	Reduced CI and weight loss, maintained of ACC, Put, and ABA levels, reduced ascorbic acid content	[139,140]
		Okra	Reduced weight loss and ascorbic acid content, increased titratable acidity	[75]
		Eggplant	Retarded chilling injury, decreased spermidine levels	[119]
		Cucumber	Reduced weight loss, decay and fruit deformation, maintenance of freshness, colour and firmness	[141,142]
		Green bell pepper	Reduced CI, weight loss, membrane leakage and LOX activity, induction of HSP from the HSP70 family	[114,118,140,143]
		Zucchini	Reduced ethylene production and ethylene gene expression, reduced H_2_O_2_ and MDA	[24]
	Ceramide coating	Green bell pepper	Maintenance of membrane integrity, reduced MDA, enhanced activity of POD, CAT, and APX	[144]
	Chitosan coating	Zucchini	Reduced CI, preservation of flesh firmness	[121]
		Cucumber	Reduced CI, electrolyte leakage and MDA accumulation, increased content of soluble solids, chlorophyll and ascorbic acid, SA, and induced activity of SOD, CAT, APX and GR	[116]
		Sponge gourd	Delayed PPO, increased content of ascorbic acid and total phenolics	[152]
Chemical	1-MCP	Green bell pepper	Delaying senescence associated with enhanced antioxidant enzyme activities	[158]
		Zucchini	Reduced fruit weight loss, respiration rate and cold-induced ethylene, reduced expression of ethylene genes	[37]
		Eggplant	Reduced weight loss and browning, and reduced of PAL, PPO and POD activity, and total phenolics	[51]
	Brassinosteroids	Green bell pepper	Decreased electrolyte leakage and MDA content, enhanced CAT, APX, and GR activities	[160]
		Eggplant	Maintenance of membrane integrity and moisture and reduced flesh browning, reduced phenolic accumulation and repressed PAL, PPO, and POD activities	[161]
	ABA	Zucchini	Delayed development of CI symptoms	[188]
	Salicyloyl chitosan coating	Cucumber	Higher total soluble solids, chlorophyll and ascorbic acid content, reduced electrolyte leakage and MDA, and induction of SOD, CAT, APX and GR	[116]
	SA and MeSA	Sponge gourd	Higher antioxidant activity reduce MDA, enhanced SOD, CAT, APX activities	[73]
	MeSA and MeJA	Green bell pepper	Increased expression of *AOX* gene	[106,178]
		Cucumber	Reduced H_2_O_2_ accumulation enhanced catalase activity	[179]
		Eggplant	Reduced ethylene production	[176]
	MeJA	Cucumber	Induced chilling tolerance by inhibiting H_2_O_2_ generation and CAT activity	[179]
	PAs	Green bell pepper	Reduction of ethylene production	[112]
		Zucchini	Induction of APX, CAT and GR activities, increased content of ascorbate, FRAP, glucose, fructose and raffinose, and reduced LOX activity	[98,99,122,180]
	Nitric oxide	Cucumber	Reduced lipid peroxidation, O_2_^−^ and H_2_O_2_ accumulation, and enhanced CAT, SOD, APX and POD activities	[95,176]
		Green beans	Shelf life extension	[183]
	6-BA	Cucumber	Increased chlorophyll, ascorbic acid, total phenolic contents, and antioxidant capacity, reduced O^•−2^, H_2_O_2_ and lipid peroxidation, increased activities of SOD, CAT, APX, GR and ATP	[89]
	CK	Zucchini	Slower deterioration and dehydration, phenolic compound accumulation, and decreased pectin and sugar solubility, delayed cell wall dismantling	[185]
	GB	Green bell pepper	Reduction in cellular leakage, MDA content, and lipid peroxidation increased activity and induced gene expression of *POD, CAT, APX,* and *GR*	[186]

**Table 3:** Transgenic plants of immature fruit species with enhanced tolerance to oxidative stress.

Species	Transgene	Effect of Overexpression/Silencing	References
Cucumber	Cassava *SOD*	Anti-oxidative cucumber as a functional cosmetic material	[190]
	Cucumber Nitric Oxide Synthase Associated 1 gene *CsNOA1*	Chilling tolerance of cucumber seedling	[179]
	*Arabidopsis CBF1*	Protection against chilling stress in cucumber leaves	[199]
	Cucumber Mitogen-activated protein kinase gene *CsNMAPK*	ROS scavenge and osmotic adjustment in cucumbers under salt stress	[204]
	Cucumber *Inducer of CBF expression 1 (ICE1)*	Enhanced chilling tolerance	[200]
	Cucumber *APX*	Alteration in ascorbate and glutathione redox states and increased sensitivity to ozone-induced oxidative stress in tobacco	[192]
Eggplant	Eggplant *APX* (*SmAPX*)	Greater resistance to flooding and less oxidative injury in *Arabidopsis* and rice	[193,194]
	Eggplant *SmMYB1* gene encoding a R2R3 MYB transcription factor	Increasing anthocyanin content in different organs, including fruit peels, and enhanced tolerance to seedling freezing stress	[201]
Bell pepper	Pepper LOX gene *CaLOX1*	Reduction of lipid peroxidation and H_2_O_2_, associated with high salinity and drought stress tolerance, and defence against pathogens in *Arabidopsis*	[196,197]
	Pepper PX gene *CaPO2*	Drought and oxidative stress tolerance in *Arabidopsis*	[195]
	Pepper *S*-adenosylmethionine decarboxylase gene *SAMDC*	Reduced ROS production and drought tolerance in *Arabidopsis*	[198]
	*Capsicum annuumRECEPTOR-LIKE KINASE 1* (*CaRLK1*)	Supress plant cell death by increasing concentration of superoxide anion	[203]
	Tomato *Cu*/*Zn SOD*	Increased resistance to oxidative damage and improved shoot regeneration in pepper	[191]
	Pepper ankyrin repeat domain zinc finger transcription factor gene *CaKR1*	Reduced levels of free oxygen radicals. Enhanced tolerance to salinity and oxidative stress in tomato	[63]
	Pepper ascorbate peroxidase-like 1 gene *CaPOA1*	Enhances tolerance to oxidative stress and pathogens in tobacco	[205]
Sponge gourd	*Luffa cylindrica ASCORBATE PEROXIDASE* (*LcAPX*)	Enhanced flood tolerance in transgenic *Arabidopsis* plants by reducing H_2_O_2_ and MDA	[193]
